# Stimulating solidarity to improve knowledge on medications used during pregnancy: A contribution from the ConcePTION project

**DOI:** 10.1186/s12910-023-00924-x

**Published:** 2023-06-27

**Authors:** Marieke J Hollestelle, Rieke van der Graaf, Miriam CJM Sturkenboom, Johannes JM van Delden

**Affiliations:** 1 Department of Bioethics and Health Humanities, Julius Center for Health Sciences and Primary Care, University Medical Center Utrecht, Utrecht University, Utrecht, The Netherlands; 2 Department Data science & Biostatistics, Julius Center for Health Sciences and Primary Care, University Medical Center Utrecht, Utrecht University, Utrecht, The Netherlands

**Keywords:** Solidarity, Pregnant people, Learning Healthcare Systems, Empowerment, Ethics, Real-World Data

## Abstract

**Background:**

Pregnant people have been overlooked or excluded from clinical research, resulting in a lack of scientific knowledge on medication safety and efficacy during pregnancy. Thus far, both the opportunities to generate evidence-based knowledge beyond clinical trials and the role of pregnant people in changing their status quo have not been discussed. Some scholars have argued that for rare disease patients, for whom, just like pregnant people, a poor evidence base exists regarding treatments, solidarity has played an important role in addressing the evidence gap. This paper explores whether and how the enactment of solidarity among pregnant people can be stimulated to help address the poor evidence base on medications used during pregnancy.

**Method:**

We use the concept of solidarity formulated by Prainsack and Buyx and enrich their concept by providing an account for stimulating the enactment of solidarity. Then we apply this account to the case of pregnant people who use medication.

**Results:**

Solidarity means enacted commitment on the part of an individual to assisting others with whom the person recognizes a similarity in a relevant respect. Although solidarity cannot be imposed, we argue that the empowerment of people is a crucial concept in understanding how solidarity can be stimulated. Empowerment in the context of pregnant people means creating awareness about their status quo, explaining how scientific research can help close the knowledge gap, and how pregnant people can themselves contribute. In particular, how pregnant people can contribute to the collection of health data to strengthen the evidence base for medications used during pregnancy.

**Conclusions:**

We conclude that acting in solidarity can help change the status quo for pregnant people. Furthermore, we argue that the empowerment of pregnant people and other relevant stakeholders is a way to stimulate the enactment of solidarity. The process of empowerment starts by raising awareness about the lack of evidence on medications used during prengnacy and by explaining to pregnant people how they can contribute to changing the way knowledge is being generated by, for example, sharing data on the health effects of medications.

## Background

Although the inclusion of pregnant people in clinical research has been widely promoted over the last decade (see Table 1), the evidence base for medication use during pregnancy remains poor. Drug manufacturers hesitate to conduct clinical trials with pregnant people, and pregnant people hesitate to participate in clinical trials, because of a fear of risks for the developing fetus [1–3]. Medications diethylstilboestrol (DES) and thalidomide are often mentioned as examples of tragedies that have strengthened the precautionary attitude towards the inclusion of pregnant people in clinical research. Between 1938 and 1971, DES was prescribed to an estimated 1.5 to 3 milion pregnant people to prevent miscarriage. The drug was later found to be ineffective and linked to several harmful complications for the offspring [4, 5]. In the late 1950s, thalidomide was prescribed to pregnant people for nausea without prior testing, resulting in unforeseen teratogenic effects and severe birth defects in over 10,000 children [6]. Although neither tragedy involved clinical research, they had a significant impact on the research community’s already protectionist approach towards pregnant people. Currently, 95% of medication labels (including vaccines, medication for obstetric and non-obstetric illnesses and conditions, and prescribed and over-the-counter medication) do not provide information on the safe use during pregnancy [7, 8]. Pregnant people and their healthcare professionals (HCPs) often face making treatment decisions based on limited evidence, which sometimes mistakenly leads to not taking medication or discontinuing treatments, which can have adverse effects on both the pregnant person and the developing fetus. Even less information is available about the exposure of the newborn to the medication through lactation. With that, the lack of knowledge on medication safety and efficacy does not only affect women but also transgender men and gender diverse people. Therefore, this paper will refer to pregnant people [9].

**Table 1:** Overview of initiatives and guidelines on the inclusion of pregnant people in clinical research

Initiative or guideline	Description	Link to website
The Second Wave Initiative 2009	The Second Wave Initiative is a collaborative academic effort from the United States that aimed to identify, develop, and advance ethically and scientifically responsible solutions for increasing the knowledge base for the treatment of pregnant people who have medical conditions.	https://www.secondwaveinitiative.org
PHASES 2016	Pregnancy and HIV/AIDS: Seeking Equitable Study (PHASES) seeks ethical solutions to advance research at the intersection of people’s reproduction and HIV prevention, treatment, and management. PHASES is an interdisciplinary, research-driven project funded through the U.S. National Institute of Allergy and Infectious Diseases of the National Institutes of Health and collaborates with international leaders in different fields across the world.	http://www.hivpregnancyethics.org
United States Task Force on research specific to pregnant and lactating women (PRGLAC) 2018	The 21st Century Cures Act established PRGLAC to advise the Secretary of Health and Human Services (HHS) on gaps in knowledge and research on safe and effective therapies for pregnant and lactating people.	https://www.nichd.nih.gov/about/advisory/PRGLAC
PREVENT 2018	Pregnancy Research Ethics for Vaccines, Epidemics and New Technologies (PREVENT) has developed concrete, actionable, consensus-driven ethics guidance on how to equitably include the interests of pregnant people and their offspring in vaccine research and development for priority pathogens and emerging epidemic threats. PREVENT is led by researchers from the United States, with external contributions from international experts.	https://bioethics.jhu.edu/research-and-outreach/projects/prevent/
CIOMS International Research Ethics Guidance (guideline 19) 2016	The Council of International Organizations and Medical Sciences (CIOMS) provides guidance to a number of pressing issues in research ethics, including research with pregnant people. CIOMS represents a substantial proportion of the international medical scientific community through its member organization across the world.	https://cioms.ch

There are strong ethical reasons to change the way evidence is currently being generated and disseminated. Given the vast availability of real-world data on medication prescriptions and health outcomes, generating evidence by learning from previous and current medication use through a Learning Healthcare System (LHS) could be an alternative strategy. In an LHS, clinical practice and research are integrated in such a way that they can support each other and accelerate research and outcomes for patients and their physicians and make the implementation of new insights in clinical practice easier [10]. Most pregnant people take at least one medication during pregnancy [7], and numerous medications are routinely used safely and effectively in pregnancy; however, we do not yet systematically learn from these experiences. There are many databases across the world that collect or have access to unique and relevant data. None of these databases was designed to cover all aspects needed to evaluate (long-term) efficacy and safety of medications used during pregnancy or to function as a meta-registry. Transforming the available evidence base for pregnant people by creating and operating within an LHS that utilizes real-world data to generate evidence reliably could be a solution. Such a system could stimulate informed decision-making regarding treatments for pregnant people [11].

To be able to utilize real-world data in an LHS, pregnant people need to support this system change. Interestingly, instead of thinking about ways to change the system of knowledge generation altogether, the focus has been, until now, on the role of individual stakeholders, such as research ethics committees, researchers, funding agencies, manufacturers, pharmacologists, and guideline committees to safeguard the interests of pregnant people in clinical research [12]. As a result, the role of pregnant people in changing the status quo and the opportunities to generate evidence-based knowledge beyond clinical trials have not been explored. Moreover, there is little demand from within pregnant people acting as a community for a systemic change [13].

From the literature, we know that solidarity plays an important role amongst rare disease patients, for whom, just like pregnant people, a poor evidence base regarding medications exists [14, 15]. It has been argued that solidarity among rare disease patients strengthened their role in shaping the research agenda and allowing them to share knowledge, experiences, and resources to achieve progress [14, 16]. Although the comparison between the group of rare disease patients and pregnant people is limited, the success from rare disease patients indicates that solidarity may be a key tool in engaging pregnant people in closing the knowledge gap. Moreover, in order to be successful, individuals might need to be encouraged to rely on solidarity to achieve progress.

In this paper, we investigate whether and how we can engage pregnant people in closing the knowledge gap by stimulating the enactment of solidarity on the part of pregnant people. This paper does not address whether solidarity is (always) morally desirable or if solidarity is even morally required because our focus is on understanding whether and how it is possible to stimulate the enactment of solidarity. Our aim is not to develop a new concept of solidarity but to apply the existing philosophical literature on solidarity to the situation of pregnant people using medications. In this paper, we first present a summary of the general discussion on solidarity. We will draw primarily on the concept of solidarity developed by Barbara Prainsack and Alena Buyx (2017), who have undertaken an extensive analysis of solidarity in the field of bioethics. We develop their concept of solidarity by providing a perspective on how to stimulate the enactment of solidarity amongst groups who are not yet unified or aware of their shared problem. Lastly, we apply solidarity in the context of pregnant people and address the need to provide information to pregnant people about the poor evidence base problem to stimulate their engagement on the basis of solidarity in, for example, an LHS. We want to emphasize that we do not place the responsibility of changing the status quo regarding the evidence base on medication safety in pregnancy on pregnant people. The lack of scientific knowledge is not their fault, but we believe they could be part of the solution.

## Solidarity in bioethics

The concept of solidarity is receiving increasing attention in (bio)medical ethics. In addition to a special issue in the journal *Bioethics* in 2012, more researchers are exploring the role of solidarity in bioethical issues. For example, solidarity in the context of medical research involving humans [17], big data, machine learning and artificial intelligence [18], and organ donation [19]. A systematic analysis of the concept and definition of solidarity is beyond the scope of this paper and we therefore provide only a brief summary. When surveying the literature on solidarity in Bioethics, scholars are in agreement that it is a complex multi-faced concept that can be used in many different ways [20–22]. The term “solidarity” has been mostly theorized in political contexts, and there are only a few attempts at incorporating solidarity within mainstream ethical theory. According to some, this neglect results from the fact that modern ethical theory seeks universalizability and focuses on values related to individual freedom. Consequently, modern ethical theories focus on the individual and does not include references to collectivity, which leaves little space for the concept of solidarity [20, 22, 23]. According to some authors, solidarity is more suited to play a central role in contexts that necessitate collectivity, like public health ethics [24, 25].

Solidarity is a challenging concept to define and theorize. There are different views on what solidarity as a phenomenon entails. Moreover, there are different conceptualizations of what solidarity is premised on; for example, concepts of empathy, altruism or collaboration, and/or more general pro-social behaviors [26]. Ter Meulen explains that although solidarity as a moral concept often implies a sense of non-instrumental support and cooperation based on the identification with a common cause, most conceptions base solidarity on self-interest. Solidarity is often explained as individuals being prepared to serve the collective interest because they expect the same behavior of others in return when needed or when the potential gains of participating outweigh the costs to them [23].

There also is uncertainty about the role of solidarity in our normative discourse. There is genuine disagreement as to whether solidarity is a value worth pursuing or whether it can be the basis of obligations. Some authors who attempted to theorize solidarity within modern ethical theories argue that it does not have a freestanding normative power and it cannot be described as a universal principle, like justice or autonomy. Instead, solidarity is a concept that can help connect these universal or more general values with specific reasons and obligations to act [20, 27, 28]. More specifically, solidarity can help specify actions when a general (bioethical) value, i.e., justice or beneficence, does not tell us what to do or how to interpret that value in a specific situation [27, 28]. Some authors explicitly focus on the relationship between solidarity and justice, arguing that justice and solidarity are equally important and complementary values that should be considered in healthcare practices and institutions [29, 30].

Despite the ambiguity, many scholars agree that the concept of solidarity has both normative and descriptive aspects. The normative aspects refer to a disposition to act in solidarity. More specifically this relates to the moral obligation of members of a group to assist one another in various ways [28]. Actions of solidarity are described as how an individual sees what ought to be done, and how to behave towards others in a social group based on a particular identity or preference shaped by belonging to that group. This is what Dawson and Verweij call *constitutive* solidarity [22]. The descriptive aspects refer to the social practices and relationships within and amongst particular groups. Dawson and Verweij refer to the term *rational* solidarity, which they suggest arises when a collective threat, acknowledged by a group or society, requires “standing together” to avoid or minimize harm. As an example, the authors refer to social distancing as an act of solidarity during a pandemic [22]. This sense of solidarity fits more naturally with the self-interest-based notion of solidarity because of the direct benefit to the individual. Simultaneously, rational solidarity also underpins what seems to be one of the most central aspects of solidarity; that solidarity often refers to created relationships between individuals, between groups, or between individuals and groups [31]. These relationships are described as created because solidarity does not evolve naturally and is, in some instances, an artificial bond between individuals and groups. Solidarity does not have to arise between friends or people who know each other. There can be solidarity with strangers, e.g. solidarity based on some identity characteristic or common goal [32]. Jaeggi argues that the ability to form relationships of solidarity is related to the capacity to cooperate [32]. Cooperating or supporting others is seen as an important moral value. Intuitively, the relational aspect of solidarity is what draws us to the concept. A solution to the current knowledge gap on medication safety during pregnancy could be a common goal to invoke a bond of solidarity between pregnant people. However, establishing that solidarity may be of utility raises the question of what we can expect from individuals when we ask for solidarity.

In the next section, we outline solidarity as we see it having utility in addressing the problem outlined for pregnant people and turn to the work of Prainsack and Buyx (2017). Their description of solidarity attempts to bridge both the normative and the descriptive aspects of the concept to allow for a clearer concept that might have more real-world applications. Prainsack & Buyx’s understanding of solidarity gives us a descriptive concept with normative implications. In addition, it tells us what kind of connectedness or relatedness provides the basis for solidarity.

### The concept of solidarity by Prainsack and Buyx

Prainsack and Buyx understand solidarity as “enacted commitments to accept costs to assist others with whom a person or persons recognize a similarity in a relevant respect” [28]. In their conceptualization, solidarity is understood as a practice. Important elements from this definition are three-fold. First, solidarity is enacted and is not a personal disposition, a general feeling, sentiment, or attitude towards another person (i.e. empathy and altruism). Second, solidarity involves a commitment and is not something an individual does once (i.e., solidarity involves more than marching in a protest on one occasion). Third, solidarity is based on the recognition of a similarity between individuals that matters in a certain context (i.e. solidarity is distinguished from donating to a charity which is oftentimes characterized by a top-down and asymmetric relationship) [28].

Solidarity relies on the *voluntariness* of individuals to help others with whom *they* recognize a similarity in a relevant respect.While bioethical values like justice, autonomy, and beneficence are articulated in a top-down manner, solidarity, especially at the interpersonal level, emerges *bottom-up* [28]. Solidarity, in that sense, is quite fragile. The essence of solidarity is what individuals are willing to do for people with whom they share a common goal. Therefore, solidarity cannot be demanded and sanctioned in the way duties of justice can be demanded [27, 28].

According to Prainsack and Buyx, solidarity can take place on three different levels, also called the tiers of solidarity: (1) the individual level (between individuals), (2) the group level (between people who consider themselves bound together through at least one similarity, such as a shared medical condition), and (3) the institutionalized level (where solidarity is institutionalized in the shape of contracts, legal or administrative norms, such as societal welfare arrangements). Tiers 1 and 2 often exist without the solidaristic norms and provisions at tier 3, while tier 3 emerges out of solidified practices of solidarity at the interpersonal or group level [28]. Consequently, in this paper, we mainly focus on solidarity on the individual level since we aim to investigate whether there is a way for solidarity to take effect from the bottom up (for pregnant people using medications to act in solidarity with one another). Over time, the enactment of solidarity can become common among people and could transform into instances of group solidarity, where solidaristic practices are normal [28].

Having explained how we conceptualize solidarity, we now should address the matter of what we expect from individuals when we ask for solidarity. Prainsack and Buyx’s account suggests that in asking for solidarity we expect people to contribute to assisting people with whom one has something in common that matters in a specific situation, which in turn, contributes to the realization of a general bioethical value, such as justice. However, understanding this as the mechanism of change also poses a challenge: Prainsack and Buyx recognize that solidarity cannot be demanded and relies on the ability and willingness of individuals to recognize a similarity in a relevant respect and the voluntariness of them to act. However, one can imagine that people might not often recognize that they share a similarity with another person or group in a relevant respect or that they need to act, and therefore, the enactment of solidarity may need encouragement. However, if soldiarity cannot be imposed, is there a way to stimulate the enactment of solidarity? Unfortunately, the work of Prainsack and Buyx does not immediately provide an answer to that question. In their work, Prainsack and Buyx use solidarity as an explanatory concept, mainly outlining solidarity as a social practice, rather than explaining whether there is a moral obligation to stimulate the enactment of solidarity among groups for whom cooperation would likely have meaningful consequences. In what follows, we contribute to the literature by providing a mechanism by which solidarity can be encouraged: the empowerment of individuals.

### Empowerment

We argue that stimulating the empowerment of people is crucial in understanding how solidarity can be invoked. The literature on the concept of empowerment is rather large, and it is beyond the scope of this paper to provide a complete account. We understand empowerment as a process that enables people to gain (more) control over their own lives. It also involves enhanced decision-making and obtaining the ability to cooperate with others to bring about change [33–36]. Empowerment is made possible by educating people and by providing information, opportunities, and resources for people to gain knowledge and experiences while also gaining (more) control over their lives [33, 37]. If people are simply unaware of their shared situation, the vulnerability resulting from it, and the ability to act, stimulating empowerment may mean providing information, opportunities, and resources for people so they can become aware that they share a specific struggle and can choose to act. Empowerment might then stimulate the enactment of solidarity because in awareness, people can understand that they can help overcome this struggle by assisting one another and standing up together. Jaeggi has made a similar observation regarding solidarity: “the ability to act [in solidarity] is related to becoming aware that one is in the same situation in such a way, that our positions are intertwined” [32]. Jaeggi does not elaborate further upon the role of empowerment in stimulating enactment of solidarity. Nonetheless, her statement underlines how empowerment could be necessary for solidarity to exist. Especially since solidarity, according to Prainsack and Buyx, emerges bottom-up and depends upon the voluntariness of individuals to act with other people with whom they share a common goal or problem [28].

#### Empowerment of pregnant people

For pregnant people, to start the process of empowerment, we believe it is important to first raise awareness about the issue of the poor evidence base for medications used in pregnancy and the harms and risks resulting from this. Raising awareness and increasing knowledge are often mentioned as the first steps for the process of empowerment in the Health Education and Patient Empowerment literature [35–38]. Starting by raising awareness within, for example, the context of routine primary care and obstetric care could enable pregnant people to understand their shared situation, their vulnerability resulting from that situation, and the need for action to help realize justice through solidarity. Next, health literacy could be increased by explaining how scientific research can help close the knowledge gap and, accordingly, explain how pregnant people can engage and contribute to closing the knowledge gap. In this way, the enactment of solidarity could be stimulated, because it would allow pregnant people to gain experiences, skills, and knowledge which could enable them to recognize that they are in a relevant shared situation.

In this account, we need to examine what pregnant people can do to help improve their situation or the situation of future pregnant people. Establishing advocay groups specifically for pregnancy can help increase engagement among pregnant people. Although such groups are commonly formed for specific diseases, they are not as prevalent for pregnancy. Apart from unifying and hopefully being more visible in demanding a change of their status quo (being a population where there is limited evidence on the impact of medications used during pregnancy), pregnant people can also contribute to already existing initiatives. The lack of knowledge is a multi-stakeholder problem, which means that the contribution of pregnant people could also potentially influence the work of many different stakeholders and their activities. For example, to be able to learn from routinely collected health data in an LHS, there needs to be enough relevant data to analyze. To make sure that there is enough relevant data to utilize, pregnant people must be aware of data collection and data analyses to improve care and generate knowledge. Although Prainsack and Buyx argue that sharing data would not necessarily count as a solidaristic action, as it does not involve active participation or some sort of personal deliberation or investment [28], there are methods of data collection that do require a more active role of pregnant people. There are, for example, prospective cohort studies that collect data via surveys or other follow-up interventions. Another example of how pregnant people can act in solidarity is by reporting side effects of medications or treatments or other complications during all sorts of treatments for various things. In this way, medication uses and their effects can be registered, and trends can be followed, leading to further investigations on side effects. Subsequently, new insights from these studies need to benefit people within the group that made the insights possible, so that they can understand how their contribution impacts knowledge generation and informed decision-making regarding medication intake during pregnancy.

## Discussion

Thus far, this paper has addressed three different points, namely: (1) there is a lack of evidence on the impact of medications used during pregnancy, (2) despite the efforts to guide the fair inclusion of pregnant people in clinical trials, a paradigm shift is needed regarding the way knowledge is being generated, by for example transforming the field into an LHS, and (3) that through empowerment, we can stimulate pregnant people to engage in the proposed paradigm shift on the basis of solidarity. However, we also need to acknowledge a few important challenges regarding the group of pregnant people that might be relevant when considering how to invoke solidarity. In general, there is a great fear of harming the developing fetus when taking medication during pregnancy. The question is whether this fear will interfere with the ability to act in solidarity with other pregnant people. Strengthening the evidence base for medication during pregnancy also depends on actual medication intake. As long as people fear taking any medication during pregnancy, it will continue to be challenging to study medication safety and efficacy. Therefore, raising awareness should cover a wide spectrum of topics, including the topic of maternal health. However, considering almost every pregnant person takes at least one medication during a pregnancy, there is a lot of knowledge to be gained from their experiences. It is of course important to prioritize the well-being of pregnant people and not ask them to try medications for the purpose of learning from their experiences. Instead, we should encourage them to share their experiences when they have decided to take a medication during their pregnancy.

To start the process of empowerment to stimulate solidarity amongst pregnant people, the support of many other important stakeholders is necessary. Besides pregnant people, HCPs, data scientists, funding agencies, registries, and other professionals must also act in solidarity with pregnant people. Their role is crucial for raising awareness on the lack of knowledge and on the importance of scientific research, and building the right infrastructure so that people can be more involved. Organizations that collect health data during pregnancies and study medication safety and teratogens, such as academic research groups and consortia, (regional and national) pregnancy and medicine registries, teratology information service (TIS) centers, pharmacovigilance and pharmacoepidemiology centers, and pharmaceutical companies, could take multiple actions to benefit pregnant people. For example, they can improve the level of transparency and earn the trust of pregnant people regarding data collection and data use by providing understandable information about the purpose and importance of data collection. A lack of trust concerning the way organizations handle people’s data and protect their privacy might hinder actions of solidarity. Moreover, organizations could engage people in data-intensive health research, via for example social media and HCPs, to improve health data literacy, and with that, allow people to take control over their situation by, for example, choosing to participate (or not) in a cohort study or to not opt out from birth and health registries.

An example of how stakeholders can contribute and work together is the Innovative Medicine Initiative (IMI) ConcePTION consortium (2019), which is a European initiative consisting of experienced public and private organisations that collect or have access to data relatd to pregnancy, childbirth, and lactation. IMI ConcePTION aims to reorganize the importance of and to ensure access to health data in such a way that it can be transformed to generate evidence and, in turn, improve the clinical practice with new insights. This initiative aims to build an ecosystem that can better monitor and communicate the safety of medications used during pregnancy and lactation, validating and regulating workflows to hasten and optimize evidence generation across Europe. New insights will be shared in scientific publications and in a publicly available knowledge bank accessible in different languages [39]. The aims and methods of this initiative are quite similar to those on which an LHS is based. Especially an LHS that aims to generate evidence by routinely collecting and processing vast quantities of clinical and research data. This type of LHS can also be called a *comprehensive data* LHS, or a *real-time* LHS once new insights of data analyses are also directly provided at the point of care [40]. IMI ConcePTION serves as a potential concrete example of an LHS we imagine to which pregnant people could contribute by, for example, reporting adverse drug reactions to currently available local organizations that collaborate with the ConcePTION LHS. However, in order to realize and obtain the cooperation of pregnant people, stakeholders should engage in raising awareness among people and making the ecosystem accessible to pregnant people and their HCPs. It should be pointed out that this paper has not fully addressed all the ethical challenges that arise when transforming the field into an LHS. In general, an LHS challenges the current structures for evaluating care and research activities, which in turn complicates traditional safeguards such as additional protections for research participants or the responsibility of HCPs to prioritize the best interest of patients. Although it is not within the scope of this paper to respond to the ethical challenges of LHSs, future research should address these issues and provide concrete guidance for the development of an ethically responsible LHS in the field of pregnancy and lactation.

It might be challenging to encourage individual pregnant people to act in solidarity with all pregnant people, including future pregnant people. Therefore, raising awareness should also involve educating people early on in pregnancies. Particularly on the challenge of not knowing whether a medication is safe during pregnancy and on ways to help strengthen the evidence base. As mentioned in the introduction, even less information is available on newborn exposure to the medication through lactation. Ideally, the empowerment of people should not only focus on pregnancy but also on lactation to stimulate the enactment of solidarity through initiatives supporting research on lactation. These conversations can, for example, take place between primary care physicians and patients early in their pregnancy or as part of the obstetric consultations. Raising awareness among many people, including the potential partner of the pregnant person could help with normalizing actions of solidarity and even solidify into practices and norms at tiers 2 and 3.

In addition, it is important to think about how practices could be developed to educate people about the poor evidence base regarding medications used during pregnancy and to realize that ‘the group of pregnant people’ is not homogeneous in a number of ways. Furthermore, culture, religious beliefs, and perspectives considerably impact the decision-making processes of pregnant people [41]. For example, there is an ethical consensus in Western societies that treatment decisions are left solely to the pregnant person. A pregnant person’s right to determine what happens to their body has great moral weight and overpowers many other ethical considerations [41]. For people with different cultural backgrounds, religious beliefs, and perspectives, understanding the collective problem might have different moral weight, or these decision-making processes might include other people, such as certain family members, close friends or HCPs, as well. This also means that a concept of solidarity could have a different place in their set of beliefs and values, influencing the role it could have during pregnancy.

There are also meaningful differences between pregnant people who are considered healthy and pregnant people who are also managing a chronic illness or condition during their pregnancy. These groups might have different perceptions and reasons for acting in solidarity. It has even been argued that the connection between people who share the same illness or condition is stronger, and therefore, invoking of solidarity is more easily imagined too [28]. With that, people with chronic illnesses or conditions may already be connected with other patients through patient advocacy groups and share similar experiences and struggles regarding pregnancy. Consequently, it could be valuable to draw attention to the evidence-base problem as well as ways for them to contribute to closing the knowledge gap within these groups. Another aspect to consider is the fact that pregnancies take up to nine months, which is not much time for being actively involved in all sorts of research activities or for participating in an advocacy group. Pregnancy is not a disease; we must not conceptualize it as such. While it may be something that affects people’s identity in a very personal way because it is a temporary condition, it might not be something that lead people to identify with other pregnant people in the longer term as a chronic disease or condition could [28]. Perhaps we cannot expect pregnant people to commit to solidarity in the way Prainsack and Buyx argue and, instead, accept single contributions as an act of solidarity. At the same time, many pregnant people are active on social media platforms online, such as pregnancy and lactation forums [42]. On these online platforms, they share experiences with and ask questions to other people who are either pregnant or just gave birth. So, in a way, there is already some sense of recognition and solidarity which could be reinforced.

## Conclusion

This paper started from the position that so far, addressing stakeholders, such as research ethics committees, researchers, funding agencies, manufacturers, separately has not led to the much-needed change in the way evidence is being generated on the safety and efficacy of medications used during pregnancy. Therefore, we emphasize the need for a paradigm shift in which the involvement of pregnant people with the help of other stakeholders becomes more central. We believe that solidarity among pregnant people and other relevant stakeholders can help improve the situation for pregnant people regarding the evidence base problem. Furthermore, we argue that the empowerment of pregnant people is a crucial step to stimulate the enactment of solidarity on the part of pregnant people and other stakeholders. The process of empowerment starts by raising awareness on the lack of evidence on medications used in pregnancy and on how people can contribute to changing the way knowledge is currently being generated, by for example sharing their health data. Ideally, all stakeholders should feel responsible for not only raising awareness about the lack of evidence on medication safety and efficacy in pregnancy and helping pregnant people find their way in acting in solidarity, but also for helping with changing the system of developing evidence on medication safety in pregnancy.

## Data Availability

All data generated or analysed during this study are included in this published article.

## Abbreviations

HCP: Healthcare professional
LHS: Learning healthcare system
ConcePTION: Continuum of Evidence from Pregnancy Exposures, Reproductive Toxicology and Breastfeeding to Improve Outcomes Now
